# Impact of an acute care surgery service on timeliness of care and surgeon satisfaction at a Canadian academic hospital: a retrospective study

**DOI:** 10.1186/1749-7922-9-4

**Published:** 2014-01-10

**Authors:** Kerollos N Wanis, Allison M Hunter, Michael B Harington, Gary Groot

**Affiliations:** 1 Department of Surgery, College of Medicine, University of Saskatchewan, 750 Spadina Cr. E, Saskatoon, SK S7K 3H3, Canada

**Keywords:** Acute care surgery, General surgery, Program evaluation, Surgeon satisfaction

## Abstract

**Introduction:**

In January 2012 an acute care surgery (ACS) model was introduced at St. Paul’s Hospital, Saskatoon, Saskatchewan. The goal of implementing an ACS service was to improve the delivery of care for emergent, non-trauma surgical patients. We examined whether the ACS model improved wait time to surgery, decreased the proportion of surgeries performed after hours, and shortened post-surgical length of stay. We also assessed whether the surgeons working in an ACS system had higher on-call satisfaction than surgeons working in a non- ACS system.

**Methods:**

A retrospective pre-post analysis was performed using data from the Discharge Abstract Database and the Organizing Medical Networked Information database. Surgeon satisfaction was evaluated using a questionnaire that was mailed to all general surgeons in Saskatoon.

**Results:**

An ACS service significantly reduced wait time to surgery for patients with all acute general surgery diagnoses from 221 minutes to 192 minutes (ρ = 0.015; CI = 5.8-52.2). Post-surgery length of stay for patients operated on for acute appendicitis, or acute cholecystitis was not reduced. On average, patients with bowel obstruction had increased length of stay following ACS service implementation. Most surgeries in our study were performed between 16:00 hours and 08:00 hours but the introduction of an ACS significantly reduced the number of afterhours surgeries (60.0% vs. 72.6%) (ρ < 0.0001). Our survey had a response rate of 75%. Overall, surgeons on an ACS service had greater satisfaction with the organization of their call schedule than surgeons not on an ACS service.

**Conclusion:**

Introduction of an ACS service in Saskatoon has decreased wait time to surgery and reduced the proportion of afterhours emergency surgeries, with no reduction in the length of post-surgery hospital stay. Satisfaction may be higher for surgeons in an ACS service.

## Introduction

The acute care surgery (ACS) model is becoming the standard model for delivering emergency general surgery care in Canada [1]. Prior to implementation of this model, emergent surgical patients were attended to by the on-call surgeon who was simultaneously required to provide care for scheduled elective cases. Tight scheduling in elective practices made providing timely care increasingly challenging, and pushed care of emergent patients to the end of the day or during the night. This threatened patient care as well as undermined surgeon satisfaction.

ACS programs across Canada vary in their structure but share the goal of improving clinical outcomes for patients with general surgical emergencies. These programs require all general surgeons, regardless of subspecialty training, to participate in acute non-trauma surgical care for a fixed period of time (typically 7 days) while forgoing their subspecialty work [2]. Results from these services have been encouraging. Studies have demonstrated significantly reduced overall time spent by patients in the emergency department, shorter times to emergency consultation by the surgical team, reduced time to surgery, and reduced overall hospital length of stay [3-5]. In addition, a majority of surgeons feel that ACS has enabled a more manageable monthly call and a more predictable elective work schedule [6].

Australian ACS models, which are similar in structure to Canadian models, have similar results. They performed a greater proportion of operations during working hours, achieved a decreased length of hospital stay post-operatively, and had reduced complication rates for acute cholecystitis [7,8]. Furthermore, an American model with a similar structure found that ACS helped to reduce after-hours surgery and improved patient care [9]. The overall effect of an ACS system has resulted in improved time to surgery, increased the proportion of emergency procedures performed during daytime working hours, and reduced post-operative complications.

St. Paul’s Hospital in the Saskatoon Health Region adopted an ACS model starting in January 2012. In this system, one surgeon dedicates an entire week to ACS while forgoing their elective practice. This surgeon is on-site during the day and takes home-call during the evenings. There are two 17:00–08:00 shifts during the week that are covered by a second surgeon.

This study compared data collected in a pre-ACS and post-ACS time frame to determine whether the introduction of an ACS service at St. Paul’s Hospital reduced time to surgery for all emergent general surgery presentations. The post-surgery length of stay for patients presenting with acute appendicitis, acute cholecytitis, and bowel obstruction was also measured. In addition, this study evaluated surgeon satisfaction with the ACS system.

## Methods

Data extracted from the Discharge Abstract Database (DAD) and the Organizing Medical Networked Information (OMNI) databases, were retrospectively examined. These data were compared from two time periods: January 1 2011 to December 31 2011 (Pre-ACS), and January 1 2012 to December 31 2012 (Post-ACS). In addition to collecting data from St. Paul’s Hospital, we also collected data from Saskatoon’s Royal University Hospital. The Royal University Hospital does not have an ACS service. The OMNI Data includes all emergent general surgery cases performed at both Saskatoon hospitals over a two year study period. From this data, we determined the average length of time patients waited, from when surgery was booked, to when surgery was initiated. In the OMNI data, there was a total of 419 patients from St. Paul’s Hospital in the pre-ACS period and 468 in the post-ACS period. From Royal University hospital there was 446 cases in 2011 and 453 in 2012. DAD data consisted of time from surgery to time of discharge. In these data, only patients with a diagnosis of acute appendicitis, acute cholecystitis, or acute bowel obstruction were considered. In the DAD data, from St. Paul’s Hospital, there was a total 286 patients in the pre-ACS period and 294 patients in the post-ACS period.

Surgeon satisfaction was determined using a series of questions relating to quality of work, teaching, and life while on-call. A questionnaire was emailed to all surgeons responsible for general surgery call in Saskatoon. The surgeons were asked to indicate their level of agreement (strongly agree, agree, neutral, disagree, strongly disagree) to nine statements. These statements were designed to assess work satisfaction and personal satisfaction with their respective call schedules. Twelve out of sixteen (75%), of the general surgeons taking call in our health region returned the survey. The levels of agreement, described above, were converted to number values out of five. The responses were anonymous and de-identified.

Statistical analysis was performed using IBM SPSS Statistics 20 for Windows. Comparison of means was performed using student *t-*test. Proportions were compared using Chi- squared test. A ρ value less than .05 was considered to represent statistical significance. Institutional ethics approval was obtained from the University of Saskatchewan Research Ethics Board.

## Results

The OMNI database contained the wait time to surgery for 419 patients at St. Paul’s Hospital in the pre-ACS-period, and 468 patients in the post-ACS period. The average wait time to surgery decreased from 221 minutes in the pre-ACS period to 192 minutes in the post-ACS period (ρ = 0.015; CI = 5.8-52.2) (Table 1). This was compared to the OMNI database data for Royal University hospital which did not implement an ACS service. At Royal University Hospital, there were 446 cases in 2011 and 453 in 2012. During this period, the average wait time to surgery decreased from 272 minutes to 250 minutes (ρ = 0.112) (Table 1).

**Table 1:** Comparison of the average wait time to surgery for the two study periods

**Hospital**	**Average wait time to surgery (minutes)**		**p-value**
	**Pre-ACS**	**Post-ACS**	
St. Paul’s Hospital	221	192	.015
Royal University Hospital	272	250	.112

Implementation of an ACS at St. Paul’s Hospital had a significant effect on the proportion of surgeries performed after regular working hours (08:00 to 16:00). In the pre-ACS period, 304 of the 419 operations (72.6%) were performed afterhours (16:00 to 08:00). This proportion of cases decreased in the post-ACS period, as 281 of the 468 operations (60.0%) were performed afterhours. This difference was statistically significant with a ρ value less than 0.0001 (Table 2).

**Table 2:** Comparison of the numbers of surgeries performed during-hours and after-hours

**Time of surgery**	**Number of surgeries performed**		**p-value**
	**Pre-ACS**	**Post-ACS**	
During hours (08:00–16:00 hours)	115	187	<0.0001
After hours (16:00–08:00 hours)	304	281	

At St. Paul’s Hospital there were 286 patients in the pre-ACS period and 294 patients in the post-ACS period who had emergency surgery for either appendicitis, cholecystitis, or bowel obstruction. The demographic information for these patients is given in Table 3. The mean age of patients in the post ACS period was older (46.92 years, from 42.57 years) (ρ =0.001). There was no statistically significant difference in the ratio of male to female patients. As well, there was no statistically significant difference in the distribution of acute appendicitis, acute cholecystitic or bowel obstruction diagnoses. With respect to patients who underwent either appendectomy or cholecystectomy for acute cholecystitis, there was also no statistically significant difference in the post-surgery length of stay (Table 3). There was however, a statistically significant increase in post surgery length of stay for patients who were operated on for acute bowel obstruction (7.99 days pre-ACS and 12.2 days post-ACS; ρ = 0.010) (Table 4).

**Table 3:** Demographic characteristics for patients in the pre-ACS and post-ACS study groups

	**Pre-ACS**	**Post-ACS**	**ρ value**
Mean age	42.57	46.92	.001
Sex			.995
Male	140 (49.0%)	144 (49.0%)	
Female	146 (51.0%)	150 (51.0%)	
Diagnosis			.193
Appendicitis	142 (49.7%)	150 (51.0%)	
Cholecystitis	55 (19.2%)	70 (23.8%)	
Bowel obstruction	89 (31.1%)	74 (25.2%)	
**Total**	**286**	**294**	

**Table 4:** Comparison of the average post-operative length of stay for the two study periods

**Diagnosis**	**Average length of stay (days)**		**p-value**
	**Pre-ACS**	**Post-ACS**	
Appendicitis	1.78	1.69	.637
Cholecystitis	2.23	2.55	.392
Bowel obstruction	7.99	12.2	.010

The surgeons at both St. Paul’s Hospital and the Royal University Hospital were surveyed to identify their level of satisfaction with their call schedules. As shown in Table 5, the surgeons at St. Paul’s Hospital who are working in the ACS system responded with higher average satisfaction to all of the questions in our survey.

**Table 5:** Satisfaction with call schedule for surgeons in an ACS service contrasted with those in a non-ACS service

**Statements regarding satisfaction with organization of call schedule**	**ACS**	**No ACS**
**Elective practice and workload**		
1. My current call schedule allows me to focus on my elective surgical practice when not on call	3.7	2.2
2. I find the number of calls I perform monthly to be manageable	4.3	2.3
3. I find the workload while on call to be manageable	3.8	3.3
4. I feel adequately equipped to deal with the cases I encounter while on call	4.3	4.0
**Work environment**		
5. While on call, I find that there is time during the day to teach residents and medical students	3.3	3.0
6. The call organization at my hospital provides for acceptable operating room accessibility	3.7	2.0
**Personal satisfaction**		
7. I feel adequately remunerated for my work while on call	2.5	2.0
8. I am satisfied with the variety of clinical cases seen while on call	4.0	2.8
9. I am satisfied with the amount of time I can spent with my family during my on call days	2.2	1.7

Legend: Average agreement with 9 statements, on a 5 point scale from strongly disagree to strongly agree, assessing surgeon satisfaction with call schedule. The average agreement of surgeons from St. Paul’s hospital (ACS) are compared side-by-side with the average agreement of surgeons from Royal University hospital (No ACS).

## Discussion

Emergency general surgery care is provided by two hospitals in Saskatoon: St. Paul’s Hospital, and Royal University Hospital. In 2012, St. Paul’s Hospital introduced an ACS service. The intention of creating this service in Saskatoon was to improve timeliness of care, with the added benefit of improving surgeon satisfaction. An improvement in timeliness of care would be identified as a reduction in the proportion of afterhours surgery, a decrease in wait time to surgery, and a reduction in post-surgery length of stay.

In this study we had the advantage of being able to compare data for wait time to surgery between two hospitals: St. Paul’s Hospital with the ACS service and Royal University Hospital without this service. After implementation of the ACS service we were expecting that there should be a reduction in the wait time to surgery for acute general surgery cases. We defined wait time to surgery as the time period from when surgery was deemed necessary and booked to when surgery was initiated. In the year following implementation of the ACS service, the wait time was shown to be decreased by an average of 29 minutes (Table 1). Every Monday through Friday, from 12:00 h – 17:00 h there is one dedicated operating theatre reserved for acute general surgical patients. Therefore, this statistically significant reduction is a reflection of the dedicated operating room time given to the ACS service. Wait time to surgery was compared to the non-ACS, Royal University Hospital data for this same period. It was noted that there was also a reduction in wait time to surgery; however, this reduction in wait time was not statistically significant. The statistically significant decrease in wait time to surgery at St. Paul’s Hospital, but not at Royal University Hospital, is in keeping with what one would predict within an ACS system, and supports the findings of other Canadian studies [1].

Afterhours surgery is associated with increased morbidity and mortality [10-12]. One of the desired effects of an ACS service is to reduce afterhours surgery and to subsequently avoid complications. Our study supports previous findings [7] that with a dedicated ACS service, there are a greater proportion of emergency operations completed during normal work hours (Table 2).

Previous studies showed that within an ACS system there was a significant decrease in the post-operative length of hospital stay for patients who underwent surgery for appendicitis [11] or acute cholecystitis [8], but not for acute bowel obstruction [3]. Our data is not in keeping with these previous findings. As shown in Table 4, there was no statistically significant decrease in the length of stay for patients who underwent an appendectomy, or cholecystectomy. This may be explained by the fact that the pre-ACS length of stay was already short, compared to these other studies [3,8,13]. An ACS service may have an impact on post-surgical length of stay, because of hypothesized reduction in complications, and more focused care of admitted acute care patients. Since our hospital already had a short length of stay for this subgroup of surgical patients, the effect of an ACS service may not have been great enough to decrease the length of stay further, given differences in patient factors. Note that Table 3 shows an increase of age of patients over this same time period, which may be associated with higher patient morbidity. With respect to the patients admitted with bowel obstruction, the post-operative length of stay actually increased (Table 4). We do not have enough data on the bowel obstruction cohort to know how long these patients were managed conservatively, with medical treatment, before going on to surgery. It is possible that, by extending hospital stay pre-operatively, these patients are at a higher risk for developing post-op complications and hospital acquired infections. By not knowing what factors were present in the post-operative recovery period, for this group of patients, one can only speculate on why post-operative length of stay was increased.

As well as assessing the clinical value of an ACS service on patient care, we were also interested in measuring the personal impact this service has with respect to surgeon satisfaction. In this study, our survey generated a 75% response rate from surgeons both at St. Paul’s Hospital (ACS) and the Royal University Hospital (non-ACS). This response rate is similar to a prior study by Helewa, et al. [6] from which our survey was adapted. Overall, we found that the surgeons at St. Paul’s Hospital, had higher average satisfaction with statements pertaining to the organization of their call schedule. The ACS surgeons still had low average satisfaction with the amount of time they can spend with family, and their remuneration while on call. However, this was still assessed to be of a higher level of satisfaction, compared to the non-ACS surgeons.

Introduction of an ACS service has not been without some drawbacks. One potential concern for ACS surgeons relates to the inherent unpredictability of working in this system. On any given day during the ACS week, the surgeon is not guaranteed to be booking surgical cases. This has economic consequences for surgeons who have less control over their income during the on-call week. Furthermore, our system includes only one dedicated operating room theater for emergency general surgery patients. Other services can book higher priority patients at the expense of general surgery cases. An obvious area of improvement, which is supported by the findings in our study, is the dedication of more than one operating room for acute general surgery patients. This will likely further improve time to surgery for patients. Overall, the satisfaction of surgeons in our service suggests that improvements in lifestyle and patient care outweigh potential concerns.

## Limitations

Our study has a number of limitations. The patients in our post-ACS group had a significantly higher mean age than those in our pre-ACS group which may have influenced the length of stay, particularly for patients with bowel obstruction. As well, it was difficult to adequately comment on the increased length of stay for the bowel obstruction group without taking into consideration patient factors, such as pre-surgery admission time and other medical co-morbidities.

The survey which we mailed to all general surgeons in Saskatoon was not returned by 4 of the surgeons (25%). A deficiency in responses exposes our results to the possibility of non-response bias. Our conclusions, that surgeons in an ACS service are generally more satisfied than those with a traditional call schedule may be influenced by the fact that Royal University Hospital, our non-ACS centre is a trauma centre while St. Paul’s Hospital is not. The surgeon who has to deal with trauma cases may respond differently to questions regarding workload and satisfaction while on call.

## Conclusion

Introduction of an acute care surgery service at an academic Canadian center has resulted in decreased wait time to surgery for patients presenting with general surgical emergencies (ρ = 0.015; CI = 5.8-52.2 minutes). There was a statistically significant decrease in the proportion of afterhours surgeries following adoption of an acute care surgery service (ρ <0.0001). Post-surgical length of stay for patients operated on for acute appendicitis, cholecyctitis, or bowel obstruction was not decreased. Surgeons operating in an acute care surgery system report high average agreement with statements regarding satisfaction with their call schedule.

## Competing interests

The authors declare that they have no competing interests.

## Authors' contributions

KW: study design, acquisition of data, data analysis and interpretation of data, drafting of the manuscript. AH: study design, acquisition of data, drafting of manuscript. MH: study concept, revision of manuscript. GG: supervised the study concept and design, revised the manuscript. All authors read and approved the final manuscript.
